# High levels of urinary naphthalene metabolites measured in a sample of California schoolchildren: a call to expand monitoring and identify exposure sources

**DOI:** 10.3389/fpubh.2026.1789602

**Published:** 2026-04-10

**Authors:** Susan Hurley, Rosemary Castorina, Daniel Sultana, McKenna Thompson, Stephanie Jarmul, Robert B. Gunier, Anuroop Nirula, Lizbeth Ortiz-Pivaral, Peyton Jacob, Jianwen She, Josephine DeGuzman, Noehmi Garcia-Jauregui, Matt Holmes, Asa Bradman

**Affiliations:** 1 Office of Environmental Health Hazard Assessment (OEHHA), California Environmental Protection Agency, Oakland, CA, United States; 2 Environmental Health Investigations Branch, California Department of Public Health, Richmond, CA, United States; 3 Center for Environmental Research and Community Health (CERCH), School of Public Health, University of California, Berkeley, Berkeley, CA, United States; 4 Environmental Health Laboratory Branch, Center for Laboratory Sciences, Richmond, CA, United States; 5 Clinical Pharmacology Program, Division of Cardiology, Department of Medicine, University of California, San Francisco, San Francisco, CA, United States; 6 All Saints Academy of Stockton, Stockton, CA, United States; 7 Little Manila Rising, Stockton, CA, United States; 8 Department of Public Health and Health Sciences Research Institute, University of California, Merced, Merced, CA, United States

**Keywords:** children, exposure, naphthalene, polycyclic aromatic hydrocarbons (PAHs), urinary biomarkers, volatile organic compounds (VOCs)

## Abstract

**Introduction:**

Polycyclic aromatic hydrocarbons (PAHs) and volatile organic compounds (VOCs) are common air pollutants, and many are human toxicants. Naphthalene is one of the most pervasive PAHs and is highly toxic. Children are especially vulnerable to the adverse health effects associated with exposure to these chemicals.

**Methods:**

We conducted a pilot study to characterize PAH and VOC exposures to schoolchildren living in a community heavily burdened by air pollution. Sixty-nine urine samples were collected from 18 children attending a school in Stockton, CA, USA in 2021. Participants were between the ages of 5 and 13 and predominantly Hispanic (78%). Samples were analyzed for monohydroxy metabolites of four PAHs, including naphthalene, and mercapturic acid metabolites of six VOCs. Urinary PAH and VOC metabolite levels were compared to the most recent US data available for children from the National Health and Nutrition Examination Survey (NHANES), collected 3–11 years earlier.

**Results:**

Naphthalene metabolite levels were nearly three-fold higher than levels in NHANES. Levels for other PAH and VOC metabolites were similar to, or lower than, those in NHANES.

**Conclusion:**

Our results are consistent with a small, but growing, body of literature that shows elevated naphthalene exposures in Hispanic/Mexican Americans and suggests overall naphthalene exposures may be on the rise in the US and elsewhere. These exploratory results underscore the need to monitor trends and identify sources of naphthalene exposure.

## 1 Introduction

Polycyclic aromatic hydrocarbons (PAHs) and volatile organic compounds (VOCs) are broad classes of chemicals, many of which are common air pollutants and have toxic health effects (1–3). Naphthalene, a semi-volatile PAH, is one of the most abundant PAHs (4, 5) and is a recognized hazardous air pollutant (6), toxic air contaminant (7) and probable human carcinogen (1). Children are especially vulnerable to adverse health effects due to their developing respiratory and immune systems, higher inhalation rates per body weight, and increased exposure during critical periods of growth and development (8–10).

In 2017, California passed Assembly Bill 617 (11) with the goal of reducing air pollutant exposures and associated health risks in California communities disproportionately impacted by air pollution. In support of these efforts, we initiated the Stockton Air Pollution Exposure Project (SAPEP) at an elementary school in Stockton, CA, USA. Located in California’s Central Valley, Stockton is burdened by some of the worst air pollution in the state (12) and its residents may experience other chemical and social stressors that cumulatively impact their health (13). Launched in 2021 as a pilot study, SAPEP collected air quality data and measured PAH and VOC metabolites in children’s urine to characterize exposures and help evaluate the effectiveness of school air filtration at reducing exposures (14). Evaluation of the air quality data showed some modest reductions in particulate and black carbon levels associated with changes to the school’s air filtration (15, 16); however, we did not observe a relationship between air filtration and changes in urinary biomarkers of PAHs and VOCs (15), in part due to the small sample size resulting from recruitment challenges posed by the COVID-19 pandemic.

Despite the small sample size, the metabolite data are valuable due to the scarcity of recent VOC and PAH biomonitoring data, especially in US children. Presented here is a summary of PAH and VOC urinary metabolite levels measured in SAPEP participants, with a comparison to the most recent data from US child participants in the National Health and Nutrition Examination Survey (NHANES) (17), collected 3–11 years prior to SAPEP.

## 2 Materials and methods

SAPEP is a project of the California Environmental Contaminant Biomonitoring Program (Biomonitoring California), a multi-departmental program that includes the California Department of Public Health (CDPH), the Office of Environmental Health Hazard Assessment (OEHHA), and the Department of Toxic Substances Control. Led by OEHHA, SAPEP was conducted in collaboration with the University of California (UC) Berkeley, Merced and San Francisco campuses, Little Manila Rising (a Stockton-based social and environmental justice organization), and administrators from the school study site.

### 2.1 Study site and participants

The study was conducted at an elementary school that serves approximately 90 children enrolled in kindergarten through eighth grade. The school draws families from Stockton and other nearby communities in California’s Central Valley. It is located in a residential neighborhood, between two major freeways, approximately one mile downwind from the largest inland port in California. Participants were recruited with assistance from our community and school partners, through various outreach strategies including video meetings, text messaging/emails, and curbside leafletting. The original study was designed as an intervention study of school air filtration where approximately half the students were in classrooms with portable air cleaners and half were in classrooms without portable air cleaners (15). The study population was a convenience sample of students at the school; all interested students were eligible for enrollment.

Eighteen children were enrolled in the study and provided up to five urine samples each. The children’s parent/guardian completed a questionnaire about sociodemographics and potential sources of their child’s VOC/PAH exposures; they also assisted in the collection of their child’s urine (if needed).

All study instruments and activities were reviewed and approved by the State of California Committee for the Protection of Human Subjects (CPHS) with reliance obtained from the UC Berkeley CPHS. Parents provided written informed consent, and participants 7–13 years old provided assent to participate.

### 2.2 Urine sample collection

Urine samples were collected in the morning and afternoon on one school day during each of two consecutive weeks in December 2021, with most participants providing a total of four samples each (Supplementary Table 1). A total of 75 urine samples and five quality control field blanks were collected. Six urine samples from two children were excluded from analyses due to incorrect/compromised sample collection, resulting in 69 valid samples from 18 children. Urine samples were transported on dry ice, held in a residential-grade freezer for short-term storage, and delivered to CDPH’s Environmental Health Laboratory (EHL) for processing. EHL aliquoted the samples and measured specific gravity and creatinine (to assess urinary dilution). The aliquots were stored at −80° C until shipped on dry ice to UC San Francisco for analysis for PAH and VOC metabolites and cotinine (to assess tobacco-related exposures).

### 2.3 Laboratory analyses for urinary metabolites

The urine samples were analyzed for metabolites of four PAHs and six VOCs by liquid chromatography–tandem mass spectrometry (LC–MS/MS). Detailed methods to measure hydroxylated PAHs are described in Jacob et al. (18). PAH analytes included monohydroxy metabolites of fluorene, naphthalene, phenanthrene, and pyrene. Due to co-elution from the LC column, the two metabolites of naphthalene (1-naphthol and 2-naphthol) were reported together as a single value (1&2-NAP). Methods to measure VOC metabolites (mercapturic acids) are described in the supplemental materials of Jacob et al. (19). VOC analytes included metabolites of acrolein, acrylonitrile, benzene, 1,3-butadiene, crotonaldehyde, and propylene oxide. Cotinine concentrations were measured by LC–MS/MS (20). See Supplementary Table 2 for a full list of the metabolites measured. Concentrations less than the limit of quantification (LOQ) were imputed as the LOQ divided by the square root of two (21) and the data were adjusted for urine dilution using creatinine.

### 2.4 NHANES data

Information on PAH and VOC metabolites, creatinine, and demographics for children ages 5–13 (to match the age range of SAPEP participants) were downloaded from the NHANES website (17). The most recent NHANES cycle with comparable data for each analyte (ranging from 2011–2012 to 2017–2018) was used. To account for NHANES’ complex multi-stage survey design, we used sample weights, pseudo strata, and pseudo sampling units according to NHANES analytical guidelines to calculate summary statistics (22).

### 2.5 Data analysis

Prior to statistical analyses, values were log-transformed to normalize the skewed distributions. Pearson correlation coefficients between metabolites were calculated based on the average log-transformed creatinine-adjusted values for each participant. Models with a random effect for participant (to account for multiple samples per individual) were used to calculate geometric means (GMs) and intraclass correlation coefficients (ICCs). The ICC was calculated as the ratio of between-subject variance to total variance and 95% confidence intervals (95% CIs) were calculated based on a beta distribution (23). To evaluate the influence of outliers on our estimated Pearson correlation coefficients and ICCs, we conducted a “leave-one-out” analysis where we iteratively dropped the values for each of the 18 participants, and then recalculated and compared the Pearson correlation coefficients and ICCs to those generated by the full dataset.

Geometric means and ICCs were only calculated for metabolites with detection frequencies ≥65%. For comparison to NHANES, statistical differences in the GMs between SAPEP and NHANES were assessed by non-overlapping 95% CIs. To match the most recent NHANES data, these comparisons were based on the creatinine-adjusted values. To facilitate comparisons with other datasets, distributions of the unadjusted values (Supplementary Table 3) and specific gravity-adjusted values (Supplementary Table 4) are included in the Supplementary materials. To account for differences in the reporting levels between our study and NHANES, we conducted a sensitivity analysis where we repeated our comparisons to NHANES after applying the higher reporting level to the database with the lower reporting level and re-censoring the data (i.e., values below reporting level were set to the higher reporting level/sqrt of 2). Statistical analyses were performed using R version 3.6.1, STATA version 13, and SAS software version 9.4.

## 3 Results

Participants were predominantly male (72%), Hispanic (78%), and were between the ages of 5 and 13. Their parents spoke either English or Spanish, and most had at least some college education (78%). A majority of children (83%) had no recent reported exposure to cigarette smoke (Table 1).

**Table 1:** Selected characteristics of SAPEP child participants (*n* = 18) and their parents.

Characteristic	Number (%)
Child participants	
Sex	
Female	5 (28)
Male	13 (72)
Age (years)	
5–7	8 (44)
8–10	4 (22)
11–13	6 (33)
Race/ethnicity^a^	
Hispanic/Latino	14 (78)
Black/African American	4 (22)
White	2 (11)
American Indian/Alaskan Native/Native Hawaiian/Other Pacific Islander	2 (11)
Asian	1 (6)
Exposed to cigarette smoke (in the last 2 days)^b^	
Yes	2 (11)
No	15 (83)
Do not know	1 (6)
Parental participants	
Sex	
Female	14 (78)
Male	4 (22)
Preferred language	
English	16 (89)
Spanish	2 (11)
Highest education of parent/guardian	
Less than 12th grade	2 (11)
High school graduate	2 (11)
Some college/AA degree	5 (28)
College/graduate degree	9 (50)
Household Income	
$0 to <$25,000	2 (11)
$25,000 to <$50,000	4 (22)
$50,000 to $75,000	5 (28)
>$75,000	5 (28)
Prefer not to answer	2 (11)

a Number is greater than 18 because some participants reported multiple race/ethnicities.

b Based on a response in either week 1 or week 2 questionnaire.

Metabolites of fluorene, naphthalene, phenanthrene, acrolein, acrylonitrile, crotonaldehyde, and propylene oxide were found in all 18 participants. Metabolites of pyrene, benzene and 1,3-butadiene were found in 17, 13, and 1 of the 18 participants, respectively. 1&2-NAP was detected in all 69 urine samples and had a GM = 19.8 μg/g-creatinine which was at least two orders of magnitude higher than any other PAH metabolite (Table 2). Other PAH metabolites were detected in 61–99 percent of urine samples with the lowest concentration for 3-FLU (GM = 41.3 ng/g-creatinine). With the exception of benzene and 1,3-butadiene, metabolites of other VOCs were found in nearly all samples (detection frequency>88%). A metabolite of acrolein (3HPMA) was found in 100 percent of the samples and had the highest concentration (GM = 331 μg/g-creatinine).

**Table 2:** Distribution of polycyclic aromatic hydrocarbon (PAH), volatile organic compound (VOC), and nicotine urinary metabolite concentrations (adjusted for creatinine) in 69 urine samples collected from 18 SAPEP participants.

Parent compound^a^	Urinary metabolite^a^	Units	LOQ (ng/mL)	Detection frequency	GM (95% CI)^b^	Median^c^	IQR^c^
Polycyclic aromatic hydrocarbons (PAHs)							
Fluorene	1-FLU	ng/g-creatinine	0.025	60.9%	NC	25.2	<LOQ–44.4
	2-FLU	ng/g-creatinine	0.025	97.1%	119 (93.5, 151)	113	83.3–141
	3-FLU	ng/g-creatinine	0.025	85.5%	41.3 (30.2, 56.4)	32.6	23.6–65.2
Naphthalene	1&2-NAP	μg/g-creatinine	0.25	100%	19.8 (13.4, 29.1)	19.8	9.95–38.4
Phenanthrene	1-PHEN	ng/g-creatinine	0.025	98.6%	69.7 (50.6, 96.1)	68.3	42.3–108
	2-PHEN	ng/g-creatinine	0.025	76.8%	47.4 (34.1, 65.8)	46.3	29.9–69.9
	3&4-PHEN	ng/g-creatinine	0.05	88.4%	78.5 (56.9, 108)	72.2	46.1–120
Pyrene	1-PYR	ng/g-creatinine	0.025	78.3%	52.2 (37.9, 71.9)	53.0	25.6–77.8
Volatile organic compounds (VOCs)							
Acrolein	3HPMA	μg/g-creatinine	1	100%	331 (274, 398)	309	223–444
Acrylonitrile	CNEMA	μg/g-creatinine	0.5	88.4%	1.46 (1.01, 2.10)	1.75	1.03–2.51
Benzene	PMA	μg/g-creatinine	0.1	40.6%	NC	<LOQ	<LOQ–0.182
1,3-Butadiene	MHBMA-1,2	μg/g-creatinine	0.5	1.4%	NC	<LOQ	<LOQ
Crotonaldehyde	HPMMA	μg/g-creatinine	1	100%	210 (167, 264)	200	138–276
Propylene oxide	2HPMA	μg/g-creatinine	1	100%	36.7 (26.3, 51.3)	31.1	22.2–50.5
Nicotine							
Nicotine	cotinine	μg/g-creatinine	1	15.9%	NC	<LOQ	<LOQ

LOQ, limit of quantification; GM, Geometric mean; CI, Confidence interval; NC, not calculated; IQR, interquartile range (25th–75th percentiles).

a See Supplementary Table 2 for CAS numbers of parent compounds and full names of urinary metabolites.

b Geometric means not calculated for metabolites with detection frequencies <65%.

c Did not account for repeated measurements from participants.

**Figure 1:**
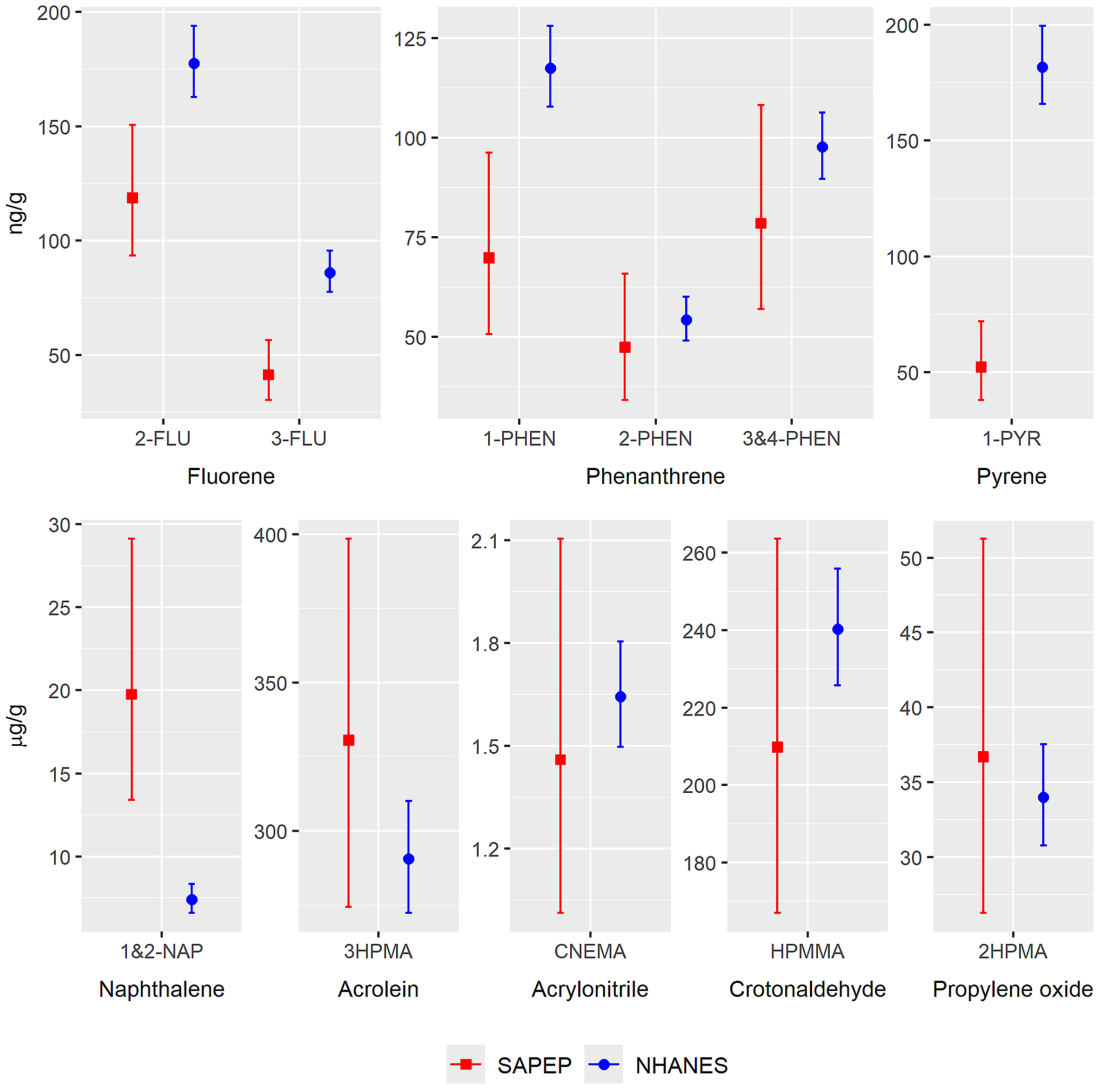
Comparison of geometric means and 95% confidence intervals for PAH and VOC urinary metabolites measured in SAPEP versus NHANES. SAPEP values are from 69 urine samples measured in 18 SAPEP participants, ages 5–13. NHANES values are for 499 + child participants ages 5–13. Comparisons were made to the most recent NHANES cycle with complete data for each metabolite. The data sources were as follows: NHANES 2017–2018: 3HPMA, CNEMA, HPMMA, and 2HPMA. NHANES 2015–2016: 2-FLU, 3-FLU, 1-NAP, 2-NAP, 1-PHEN, and 1-PYR. NHANES 2011–2012: 2-PHEN, 3-PHEN, and 4-PHEN. Comparisons were not made for the metabolites of benzene or 1,3-butadiene due to detection frequency <65% in SAPEP. Comparison was not made for 1-FLU because it is not measured by NHANES.

Compared to NHANES (Supplementary Table 5), the levels of most PAH and VOC metabolites in SAPEP were similar or lower (Figure 1). The GM of 1&2-NAP in SAPEP (GM, 95% CI = 19.8, 13.4–29.1 μg/g-creatinine), however, was nearly three times higher than that for children ages 5–13 in NHANES (GM, 95% CI = 7.4, 6.6–8.4 μg/g-creatinine). All SAPEP participants had at least one urine sample with a 1&2-NAP value above the NHANES median, and half had at least one value above the NHANES 90th-percentile. Although NHANES data show exposures are higher in Hispanic children, the 1&2-NAP geometric mean in SAPEP was also significantly higher than Hispanic children enrolled in NHANES (GM = 10.5, 95% CI = 9.1–12.1 μg/g-creatinine). Sensitivity analyses to evaluate the effect of different reporting levels between SAPEP and NHANES for some metabolites did not substantively change the results (Supplementary Figure 1).

Cotinine was detected in only 16 percent of the urine samples (from five children) and therefore could not be quantitatively included in our analysis. Our laboratory method, with a LOQ of 1 nanograms/milliliter (ng/mL) was developed for detection of active smoking but can also detect relatively high levels of secondhand smoke exposures (20). Two of the five children with cotinine detections reported recent exposures to secondhand cigarette smoke prior to urine collection. Cotinine values were generally consistent with exposures to secondhand smoke [i.e., <30 ng/mL (24)], with the exception of three values (34.3 ng/mL, 86.0 ng/mL, 55.1 ng/mL) from two children which suggested heavy secondhand smoke exposure and/or active smoking.

**Figure 2:**
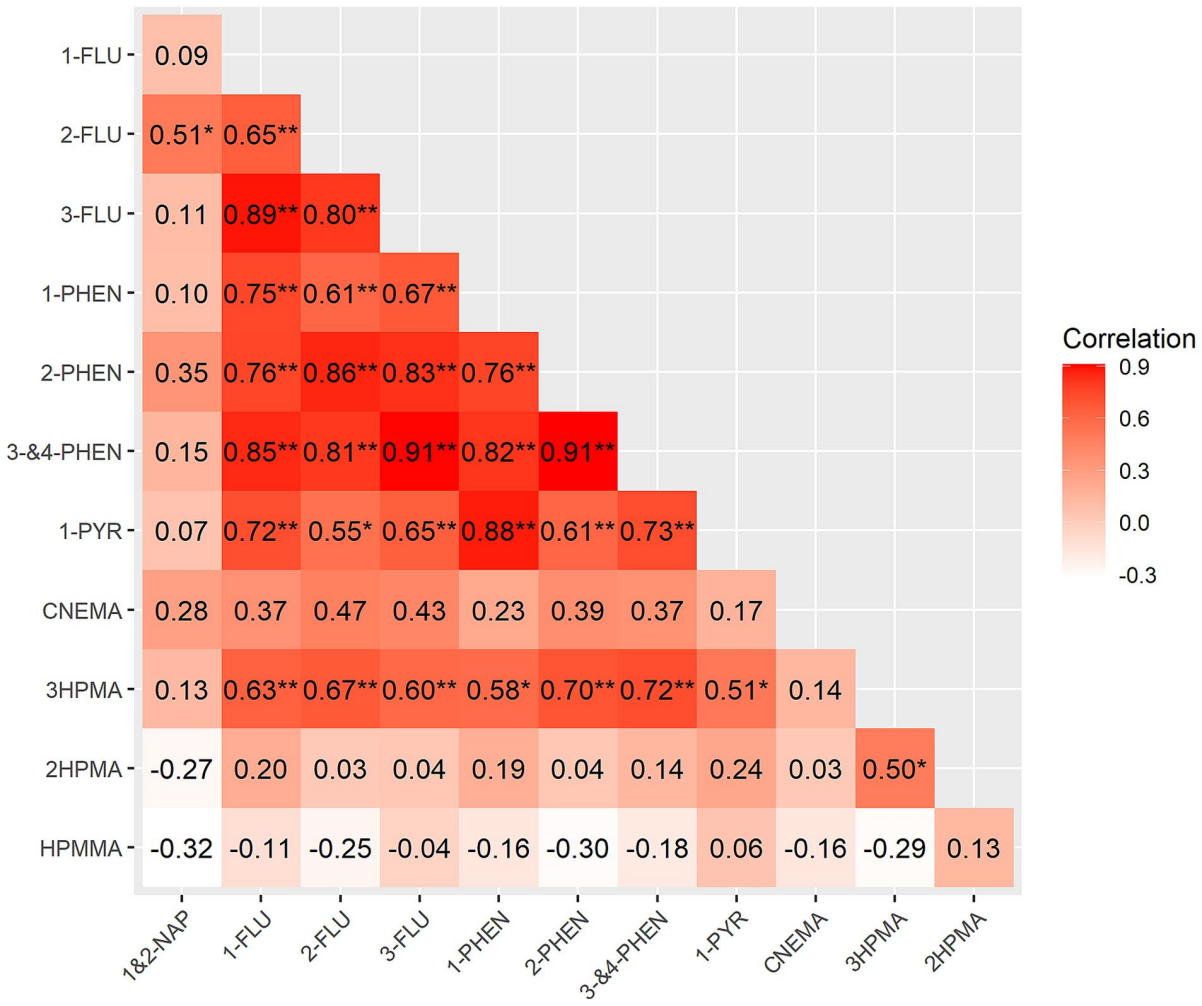
Heatmap of correlations between urinary PAH and VOC metabolites measured in 69 urine samples from 18 SAPEP participants. Pearson correlation coefficients based on the average log-transformed creatinine-adjusted metabolite values for each participant (*n* = 18), *p-value <0.05, **p-value <0.01.

With the exception of 1&2-NAP, all other PAH metabolites were moderately/highly correlated with each other, with Pearson correlations ranging from 0.55 to 0.91, *p* ≤ 0.05 (Figure 2). 1&2-NAP was also the only PAH metabolite not significantly correlated with 3HPMA (metabolite of acrolein). Our leave-one-out sensitivity analyses yielded similar correlation coefficients. Compared to the full analysis, the median difference in coefficients based on the leave-one-out analysis ranged from −0.010 to 0.003. With the exception of one iteration, the correlation coefficients from the full dataset fell within the 95% confidence intervals of the iterative leave-one-out analysis.

ICCs for the PAHs (range: 0.39–0.89) were generally higher than for the VOCs (range: 0.18–0.58) (Supplementary Table 6). The ICC for 1&2-NAP was 0.89, indicating that between-subject variance was substantially greater than within-subject variance. Our leave-one-out sensitivity analysis yielded ICCs very similar to the original ICCs generated from the full dataset, with median ICCs differing from the original ICC by <2.2% and a maximum absolute difference of 0.11.

## 4 Discussion

### 4.1 Key findings

Mirroring US national data, this pilot study demonstrated nearly ubiquitous exposure to several common PAHs and VOCs in a convenience sample of predominantly Hispanic children attending a school in California’s Central Valley. Despite its location in an area heavily impacted by air pollution, our study found that most PAH and VOC urinary metabolite levels were similar to, or lower than, those reported nationally by NHANES. In contrast, the level of 1&2-NAP was nearly three-fold higher than NHANES. Also notable was 1&2-NAP’s high ICC, which in light of its short half-life, suggests naphthalene exposures may have been continuous rather than episodic. The lack of correlation between 1&2-NAP and the other PAH metabolites suggests a distinct exposure source.

### 4.2 Prior research

The comparison of our 1&2-NAP data to national US data is based on NHANES samples collected in 2015–2016, approximately 5 years prior to the conduct of SAPEP. Biomonitoring data for naphthalene in children, especially in North America, is sparse, with very little data collected more recently than the NHANES data that we used for comparison. Our levels are several times higher than those reported in two studies of girls living in the San Francisco Bay Area of California (25, 26) and about twice as high as those reported in a population of children born to Dominican and African American mothers living in New York City (27). While one of these studies was conducted in 2017–2020 (26), the other two were based on samples collected prior to 2015. Both California studies reported that levels were elevated in Hispanics (25, 26).

Our naphthalene metabolite levels are also markedly higher than those reported by biomonitoring surveillance programs in other countries. SAPEP 1&2-NAP levels (GM = 19.8 μg/g-creatinine) are approximately four times higher than those reported by both the 2014–2015 Canadian Health Measures Survey [GM = 4.90 μg/g-creatinine for children age 6–11 (28)] and the 2014–2017 German Environmental Survey for Children and Adolescents GerES V [GM = 4.4 μg/g-creatinine for children 3–17 years old (29)]. With the exception of two studies conducted in heavily industrialized areas of Asia which reported levels similar to ours (30, 31), SAPEP naphthalene metabolite levels are strikingly higher than those reported in other exposure/epidemiologic studies of children outside the US (32–44). It is important to note that most of these studies were conducted during a similar, or earlier, time period than the NHANES data to which we have compared our levels in the current analysis.

The need for more contemporary data on naphthalene exposures is underscored by trend analyses of biomonitoring data that have shown naphthalene exposures to be increasing during the first two decades of this century in the US (27, 45, 46) and elsewhere (42, 47), particularly among children (45, 48) and Mexican Americans/Hispanics (45, 48).

### 4.3 Interpretation

Without more contemporaneous US biomonitoring data, it is difficult to discern whether the elevated 1&2-NAP concentrations in our study are reflective of increases in exposure among the general population or indicative of exposures that are unique to our study population. Regardless, our results indicate the need for further monitoring of trends and investigation of exposure sources.

Similar to other PAHs, dietary ingestion and inhalation of contaminated air (both ambient and indoor) are believed to be the predominant naphthalene exposure pathways among the general population (1, 49–51). Among those who smoke cigarettes or live in smoking households, tobacco smoke is a major exposure source (1, 49–51). In urban areas, motor vehicle emissions are an important source of naphthalene air contamination, both outdoors and through infiltration of ambient air into indoor environments (1, 49).

Since SAPEP was a small pilot study not specifically designed to focus on naphthalene, we were not able to fully evaluate these exposure sources. We do, however, have data that qualitatively suggest our findings are not being driven by exposures from tobacco smoke or ambient air. Cotinine measurements and questionnaire responses indicate that tobacco exposures among SAPEP participants were minimal. Indoor and outdoor naphthalene air concentrations collected at the school concurrent with urine collection were not unusually high (*personal communication, Dr. Elizabeth Noth, UC Berkeley*), falling within the range of typical non-smoking residences [0.18–1.7 micrograms/cubic-meter (μg/m^3^)] and of outdoor urban environments [0.02–0.31 μg/m^3^ (49)]. High urinary 1&2-napthol levels were observed in both weeks of the study, despite drastically lower air concentrations of naphthalene during the second week (likely due to a major rainstorm). Furthermore, it is notable that while PAHs (including naphthalene) and acrolein (a VOC) are constituents of motor vehicle emissions, 1&2-NAP was not correlated with 3HPMA (a metabolite of acrolein) but the other PAH metabolites were. Finally, while naphthalene shares many common sources with other PAHs (2, 52), metabolite levels of the other PAHs in our study were similar to, or lower than NHANES. These lower levels are consistent with overall declines in traffic-related air pollution levels (53, 54) and declines in tobacco use in the US over the last few decades (55) that may be more pronounced in California (56) compared to the rest of the nation. As a whole, this set of observations suggests the importance of other exposure sources beyond tobacco smoke and ambient air in our study.

Unlike other PAHs, naphthalene is used as a fumigant, deodorizer, and insecticide in consumer, home furnishing, and building products (49, 50, 57). Use of these products has been linked to household exposures (1, 50, 57). While the sale of products containing naphthalene was banned in California over 20 years ago (57), it is possible that California residents may still have access to such products through vendors outside of the state. Since SAPEP was not specifically designed to focus solely on naphthalene, we did not collect detailed information on these types of products.

Our findings suggest the source(s) of naphthalene exposure were pervasive, on-going, and distinct from the other PAHs. The elevated levels were not limited to just a few children; over half of the participants had at least one value above the NHANES 90th percentile. Naphthalene exposures were likely not unique to the school environment since elevated levels of 1&2-NAP concentrations were found in urine samples collected both before and after school. Participants’ residences were scattered across an area with an approximate 8-mile radius and with no apparent geographic clustering, making it unlikely that a point source of environmental contamination was responsible for the high naphthalene exposures. The high ICC for 1&2-NAP suggests exposures were continuous, especially since most urine samples were collected at time intervals greater than the urinary excretion half-life [ranging from 2.4–9.8 h (58)]. Finally, while the other PAH metabolites were highly correlated with each other, the naphthalene metabolites were not correlated with the other PAH metabolites, indicating naphthalene exposures may be arising from a distinct source.

### 4.4 Limitations

These exploratory findings are from a small convenience sample not designed to be representative of the underlying population and could be due to chance. Our 1&2-NAP levels, however, are substantially higher compared to levels reported in dozens of populations spread all over the world. This trend suggests that naphthalene exposures in SAPEP are truly elevated. The lack of contemporaneous data from other study populations makes it difficult to determine whether rising background naphthalene exposures in the general public or specific exposure sources unique to our study population are the cause of our higher 1&2-NAP levels.

It is also important to note that because of co-elution, 1-NAP and 2-NAP were reported together as a single value. Prior sensitivity analyses of the LC–MS/MS method that was used in our study [Figure 8 in (18)] indicated that the sensitivity was much lower for 1-NAP than for 2-NAP (by a factor of approximately 20). While this suggests that our 1&2-NAP value should be a fairly accurate estimate of 2-NAP levels, the co-elution of 1-NAP and 2-NAP presents some limitations in interpretation. 2-NAP is a specific metabolite of naphthalene but 1-NAP is a metabolite of both naphthalene and carbaryl. High levels of 1-NAP have been shown to correlate with carbaryl insecticide exposures (59). Agricultural carbaryl applications in California do not, however, typically occur during the winter months. Furthermore, the sale of carbaryl-based consumer products was banned in California in 2020, a year before SAPEP was initiated. While unlikely, we cannot dismiss the possibility that our elevated levels of 1&2-NAP are at least partially due to carbaryl exposure.

Because one of the original primary aims of SAPEP was to evaluate the effectiveness of air filtration at one school, the spatiotemporal scope of our study was limited by design. While NHANES samples are collected from both urban and rural areas year-round, the SAPEP samples were collected at one school on 2 days over the course of 2 weeks during the winter when ambient levels of PAHs are typically elevated in the Central Valley (53). While these differences may have confounded our comparisons to NHANES, our levels are also higher compared to other populations living in diverse places and measured over a variety of time frames.

It was beyond the scope of our analyses to evaluate the specific health effects of naphthalene exposures in our study population. As a small pilot study of PAH and VOC exposures, we did not collect sufficient health outcome data to evaluate potential health effects. Furthermore, the lack of human biomonitoring health-based guidance values for 1&2-NAP precludes our ability to translate the levels measured in our study with specific human health effects.

Finally, the limited information on diet and other potentially important sources of naphthalene exposure hindered our ability to pinpoint exposure sources. It is precisely because of this limitation that more monitoring and investigation of exposure sources is warranted.

### 4.5 Future directions

Biomonitoring California is conducting additional studies in California’s Central Valley to examine PAH exposures. Results from these studies may help elucidate exposure sources and pathways relevant to our SAPEP population. However, additional studies are needed to clarify temporal trends and identify current exposures in other populations and regions. Given the carcinogenic and toxic properties of naphthalene, such research should be a public health priority. Future work should consider novel exposures sources, especially those arising from consumer products (such as cleaning, personal care, and pest control products), building materials, and home furnishings (1, 49–51). Furthermore, such investigations should include efforts to better understand “off label” uses of these products, often promoted on social media (60–62), for which anecdotal accounts exist but quantitative assessments are lacking (49, 50, 57). Such information is necessary for identifying important sources of exposure which is critical for crafting legislation to reduce exposure and protect health. Finally, it is worth noting that our study population was predominantly Hispanic. In light of other biomonitoring data that suggest naphthalene exposures are higher among Hispanics/Mexican Americans (25, 26, 45, 46, 48, 63), investigations focused on these populations should be a priority, given the potential for health impacts.

## Data Availability

The datasets presented in this article are not readily available. Because of the small sample size and the sensitive nature of the questions asked in this study, survey respondents were assured raw data would remain confidential and would not be shared. Requests to access the datasets should be directed to the corresponding author.
