# Supplementary material for: High levels of urinary naphthalene metabolites measured in a sample of California schoolchildren: a call to expand monitoring and identify exposure sources
Source: Front Public Health. 2026 Apr 10;14:1789602. doi: 10.3389/fpubh.2026.1789602 (PMC13106563; doi:10.3389/fpubh.2026.1789602)
Supplement: Supplementary file 1 [file Image_1.pdf]

Supplemental Figure 1. Sensitivity analysis: comparison of geometric means and 95% confidence intervals for PAH and VOC metabolites measured in SAPEP versus NHANES, adjusting for differences in reporting levels*

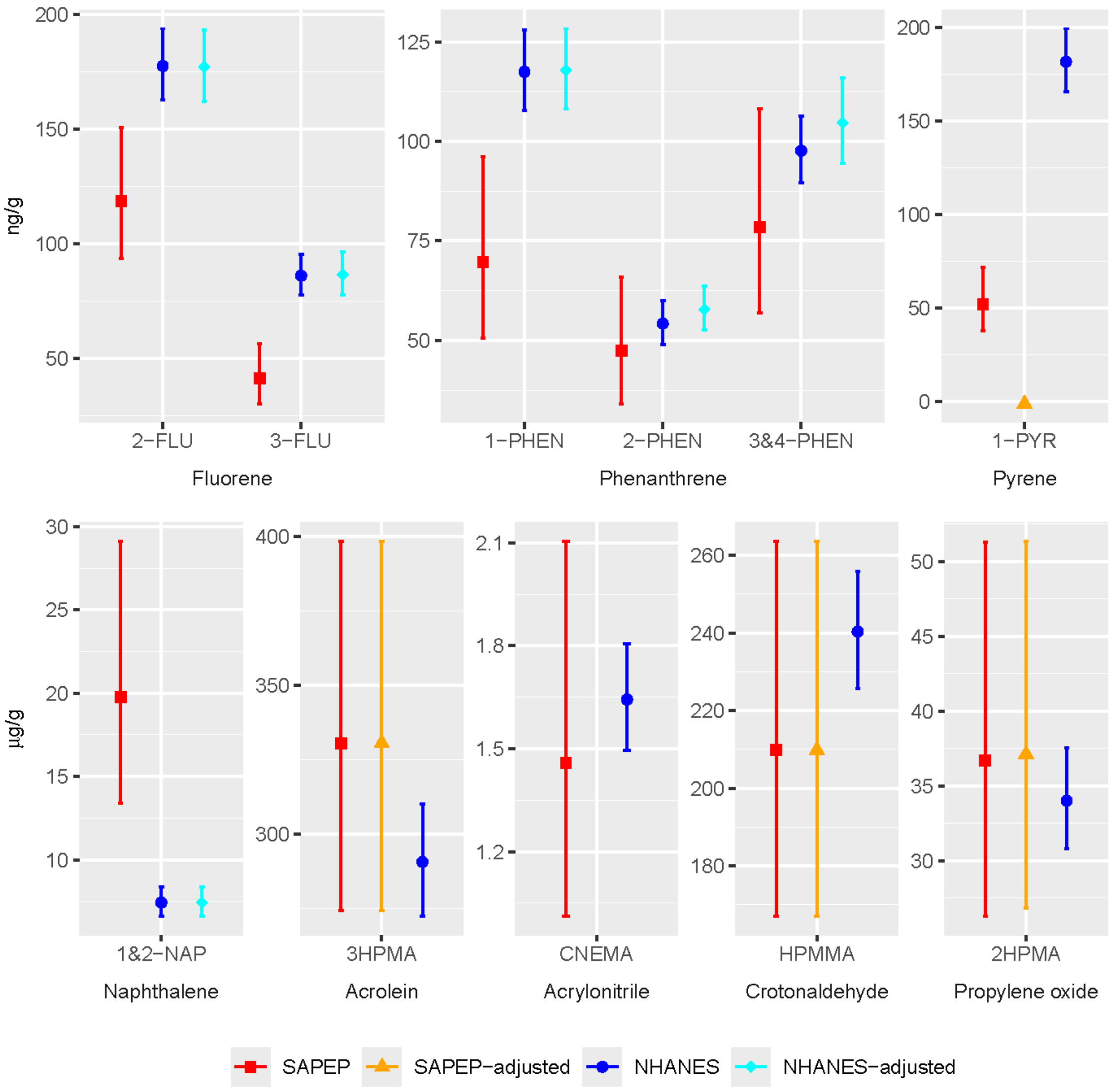

* NOTES: For 2-FLU, 3-FLU, 1-PHEN, 2-PHEN, 3&4-PHEN, and 1&2-NAP the reporting level was higher for SAPEP (Table 2) than for NHANES (Supplemental Table 5). Therefore, the NHANES GM and 95% CIs were adjusted by censoring the NHANES data to SAPEP's reporting level. For 1-PYR, 3HPMA, HPMMA, and 2HPMA the reporting level was higher for NHANES (Supplemental Table 5) than for SAPEP (Table 2). Therefore, the SAPEP GM and 95% CIs were adjusted by censoring the SAPEP data to NHANES' reporting level. An adjusted GM could not be calculated from 1-PYR because application of the higher NHANES reporting level resulted in a detection frequency < 65%. For CNEMA, the reporting levels in SAPEP and NHANES were the same; therefore, no adjusted GMs were calculated. SAPEP values are from 69 urine samples measured in 18 SAPEP participants, ages 5-13; NHANES values are for 499+ child participants ages 5-13. Comparisons were made to the most recent NHANES cycle with complete data for each metabolite. The data sources used were as follows: NHANES 2017-2018 for 3HPMA, CNEMA, HPMMA and 2HPMA; NHANES

2015-2016 for 2-FLU, 3-FLU, 1-NAP, 2-NAP, 1-PHEN, and 1-PYR; NHANES 2011-2012 for 2-PHEN, 3-PHEN, and 4-PHEN.
