# Supplementary material for: High levels of urinary naphthalene metabolites measured in a sample of California schoolchildren: a call to expand monitoring and identify exposure sources
Source: Front Public Health. 2026 Apr 10;14:1789602. doi: 10.3389/fpubh.2026.1789602 (PMC13106563; doi:10.3389/fpubh.2026.1789602)
Supplement: Supplementary file 2 [file Table_1.docx]

## Supplemental Table 1. Urine collection dates and times for 69 samples collected for each SAPEP participant (n=18)

| Participant | Date and Time | | | | | | | | No. Urine Samples |
| --- | --- | --- | --- | --- | --- | --- | --- | --- | --- |
|  | 12/6/2021 | | 12/7/2021 | | 12/13/2021 | | 12/14/2021 | |  |
|  | AM | PM | AM | PM | AM | PM | AM | PM |  |
| 1 | 7:25 | 3:01 |  |  | 7:08 | 3:01 |  |  | 4 |
| 2 | 7:15 | 3:00 |  |  | 7:10 | 2:53 |  |  | 4 |
| 3 | 6:15 | 3:30 |  |  | 6:10 | 2:57 |  |  | 4 |
| 4 | 6:51 | 3:08 |  |  | 6:50 |  | 7:02 |  | 4 |
| 5 |  | 3:10 | 7:30 | 3:12 |  |  |  |  | 3 |
| 6 | 7:23 | 3:27 |  |  | 7:35 | 3:17 |  |  | 4 |
| 7 | 7:00 | 3:12 |  |  | 6:49 | 3:24 |  |  | 4 |
| 8 | 6:15 | 3:14 |  |  | 6:30 | 3:05 |  |  | 4 |
| 9 | 7:20 | 3:05 | 6:55 |  | 7:30 | 2:40 |  |  | 5 |
| 10 | 7:16 | 3:00 |  |  | 8:01 | 2:55 |  |  | 4 |
| 11 | 7:05 | 3:21 |  |  |  |  |  |  | 2 |
| 12 | 7:19 | 3:00 |  |  | 7:00 | 2:59 |  |  | 4 |
| 13 | 6:55 | 3:10 |  |  | 6:55 | 2:58 |  |  | 4 |
| 14 | 6:00 | 3:02 |  |  | 6:00 | 3:00 |  |  | 4 |
| 15 | 7:08 | 3:05 |  |  | 7:45 | 3:05 |  |  | 4 |
| 16 |  |  | 7:45 |  |  |  | 7:40 | 3:15 | 3 |
| 17 |  |  | 5:30 | 3:20 |  |  | 6:00 | 3:01 | 4 |
| 18 |  |  | 6:45 | 3:14 |  |  | 6:50 | 3:00 | 4 |
| No. Urine Samples | 14 | 15 | 5 | 3 | 13 | 12 | 4 | 3 | 69 |
