# Supplementary material for: High levels of urinary naphthalene metabolites measured in a sample of California schoolchildren: a call to expand monitoring and identify exposure sources
Source: Front Public Health. 2026 Apr 10;14:1789602. doi: 10.3389/fpubh.2026.1789602 (PMC13106563; doi:10.3389/fpubh.2026.1789602)
Supplement: Supplementary file 4 [file Table_3.docx]

## Supplemental Table 3. Distribution of polycyclic aromatic hydrocarbon (PAH), volatile organic compound (VOC), and nicotine urinary metabolite unadjusted concentrations in 69 urine samples collected from 18 SAPEP participants

| **Parent Compound*** | **Urinary metabolite*** | **Units** | **Geometric mean  (95%CI)**** | **Median^±^** | **IQR^±^** |
| --- | --- | --- | --- | --- | --- |
| **Polycyclic Aromatic Hydrocarbons (PAHs)** | | | | | |
|  | 1-FLU | ng/L | NC | 29.3 | 18.0 - 50.4 |
| fluorene | 2-FLU | ng/L | 132 (89.8, 195) | 140 | 88.4 - 219 |
|  | 3-FLU | ng/L | 46.1 (32.8, 64.9) | 39.4 | 27.1 - 66.1 |
| naphthalene | 1&2-NAP | µg/L | 21.9 (12.7, 37.9) | 24.5 | 13.6 - 57.4 |
|  | 1-PHEN | ng/L | 77.8 (58.7, 103) | 75.2 | 47.0 - 119 |
| phenanthrene | 2-PHEN | ng/L | 53.1 (36.7, 76.7) | 52.4 | 28.5 - 85.5 |
|  | 3&4-PHEN | ng/L | 87.8 (63.0, 122) | 73.0 | 51.4 - 116 |
| pyrene | 1-PYR | ng/L | 58.9 (42.3, 81.8) | 57.7 | 29.7 - 101 |
| **Volatile Organic Compounds (VOCs)** | | | | | |
| acrolein | 3HPMA | µg/L | 374 (270, 517) | 420 | 314 - 587 |
| acrylonitrile | CNEMA | µg/L | 1.66 (1.06, 2.60) | 2.45 | 1.38 - 3.28 |
| benzene | PMA | µg/L | NC | < LOQ | < LOQ - 0.180 |
| 1,3-butadiene | MHBMA-1,2 | µg/L | NC | < LOQ | < LOQ |
| crotonaldehyde | HPMMA | µg/L | 233 (152, 358) | 239 | 168 - 417 |
| propylene oxide | 2HPMA | µg/L | 41.0 (26.0, 64.7) | 43.1 | 28.3 - 71.9 |
| **Nicotine** | cotinine | µg/L | NC | < LOQ | < LOQ |

LOQ: Limit of quantification

CI: Confidence interval

NC: Not calculated

IQR: Interquartile range (25^th^-75^th^ percentiles)

* See Supplemental Table 2 for CAS numbers of parent compounds and full names of urinary metabolites

** Geometric means not calculated for metabolites with detection frequencies < 65%

**^±^** Percentiles did not account for repeated measurements from participants
