# Supplementary material for: High levels of urinary naphthalene metabolites measured in a sample of California schoolchildren: a call to expand monitoring and identify exposure sources
Source: Front Public Health. 2026 Apr 10;14:1789602. doi: 10.3389/fpubh.2026.1789602 (PMC13106563; doi:10.3389/fpubh.2026.1789602)
Supplement: Supplementary file 5 [file Table_4.docx]

## Supplemental Table 4. Distribution of polycyclic aromatic hydrocarbon (PAH), volatile organic compound (VOC), and nicotine urinary metabolite concentrations (adjusted for specific gravity) in 69 urine samples collected from 18 SAPEP participants

| **Parent Compound*** | **Urinary metabolite*** | **Units**** | **Geometric mean  (95% CI)^** | **Median^±^** | **IQR^±^** |
| --- | --- | --- | --- | --- | --- |
| **Polycyclic Aromatic Hydrocarbons (PAHs)** | | | | | |
|  | 1-FLU | ng/L | NC | 32.6 | 20.4 - 48.9 |
| fluorene | 2-FLU | ng/L | 141 (107, 184) | 127 | 88.1 - 185 |
|  | 3-FLU | ng/L | 48.9 (36.7, 65.2) | 46.3 | 31.5 - 64.2 |
| naphthalene | 1&2-NAP | µg/L | 23.3 (14.8, 36.8) | 22.0 | 12.1 - 54.7 |
|  | 1-PHEN | ng/L | 82.4 (64.5, 105) | 88.3 | 50.8 - 119 |
| phenanthrene | 2-PHEN | ng/L | 56.2 (40.5, 78.0) | 55.8 | 40.1 - 77.2 |
|  | 3&4-PHEN | ng/L | 93.0 (70.3, 123) | 84.8 | 59.7 - 122 |
| pyrene | 1-PYR | ng/L | 61.7 (48.3, 78.8) | 60.3 | 36.3 - 89.7 |
| **Volatile Organic Compounds (VOCs)** | | | | | |
| acrolein | 3HPMA | µg/L | 395 (324, 481) | 376 | 278 - 513 |
| acrylonitrile | CNEMA | µg/L | 1.74 (1.18, 2.57) | 2.17 | 1.41 - 3.10 |
| benzene | PMA | µg/L | NC | < LOQ | < LOQ - 0.210 |
| 1,3-butadiene | MHBMA-1,2 | µg/L | NC | < LOQ | < LOQ |
| crotonaldehyde | HPMMA | µg/L | 249 (189, 327) | 235 | 173 - 348 |
| propylene oxide | 2HPMA | µg/L | 43.5 (31.1, 60.9) | 37.9 | 24.8 - 74.1 |
| **Nicotine** | cotinine | µg/L | NC | < LOQ | < LOQ |

LOQ: Limit of quantification

CI: Confidence interval

NC: Not calculated

IQR: Interquartile range (25^th^-75^th^ percentiles)

* See Supplemental Table 2 for CAS numbers of parent compounds and full names of urinary metabolites

** Specific-gravity (SG) adjusted value = (unadjusted value) x (1.021 - 1) / (sample SG -1 ) where 1.021 is the median specific gravity from children 5 to 13 in the 2007-2008 NHANES cycle.

^ Geometric means not calculated for metabolites with detection frequencies < 65%

**^±^** Percentiles did not account for repeated measurements from participants
