# Supplementary material for: High levels of urinary naphthalene metabolites measured in a sample of California schoolchildren: a call to expand monitoring and identify exposure sources
Source: Front Public Health. 2026 Apr 10;14:1789602. doi: 10.3389/fpubh.2026.1789602 (PMC13106563; doi:10.3389/fpubh.2026.1789602)
Supplement: Supplementary file 7 [file Table_6.docx]

## Supplemental Table 6. Intraclass correlation coefficients (ICCs) and 95% confidence intervals (95% CIs) for metabolites of PAHs and VOCs measured in 69 urine samples from 18 SAPEP participants.

| **Urinary metabolite** | **Between subject variance** | **Within subject variance** | **Intraclass Correlation Coefficient (ICC)*** | **95% CI** |
| --- | --- | --- | --- | --- |
| **Metabolites of PAHs** | | |  |  |
| 2-FLU | 0.4031 | 0.2838 | 0.59 | 0.36 – 0.80 |
| 3-FLU | 0.6864 | 0.4879 | 0.58 | 0.35 – 0.80 |
| 1&2-NAP | 1.2309 | 0.1452 | 0.89 | 0.82 – 0.95 |
| 1-PHEN | 0.7628 | 0.3880 | 0.66 | 0.45 – 0.84 |
| 2-PHEN | 0.7260 | 0.6916 | 0.51 | 0.27 – 0.75 |
| 3&4-PHEN | 0.7066 | 0.5961 | 0.54 | 0.30 – 0.77 |
| 1-PYR | 0.6028 | 0.9623 | 0.39 | 0.14 – 0.66 |
| **Metabolites of VOCs** | | |  |  |
| 3HPMA | 0.1595 | 0.5041 | 0.24 | 0.04 – 0.55 |
| CNEMA | 0.5206 | 2.3047 | 0.18 | 0.02 – 0.49 |
| HPMMA | 0.3507 | 0.3274 | 0.52 | 0.28 – 0.75 |
| 2HPMA | 0.7881 | 0.5609 | 0.58 | 0.35 – 0.80 |
|  |  |  |  |  |
| *Most children had 4 urine samples (see Supplemental Table 1 for details). A random effects model was fit to the log-transformed creatinine-adjusted metabolite concentrations. The ICC was calculated as the ratio of between subject variance to total variance. ICCs were not calculated for 1-FLU, PMA, and MHBMA-1,2 due to detection frequencies < 65%. | | | | |
